# EEG Microstates and Its Relationship With Clinical Symptoms in Patients With Schizophrenia

**DOI:** 10.3389/fpsyt.2021.761203

**Published:** 2021-10-28

**Authors:** Qiaoling Sun, Jiansong Zhou, Huijuan Guo, Ningzhi Gou, Ruoheng Lin, Ying Huang, Weilong Guo, Xiaoping Wang

**Affiliations:** ^1^Department of Psychiatry, The Second Xiangya Hospital of Central South University, Changsha, China; ^2^National Clinical Research Center for Mental Disorders, The Second Xiangya Hospital of Central South University, Changsha, China

**Keywords:** schizophrenia, clinical symptoms, resting-state, electroencephalogram (EEG), microstates

## Abstract

Schizophrenia is a complex and devastating disorder with unclear pathogenesis. Electroencephalogram (EEG) microstates have been suggested as a potential endophenotype for this disorder. However, no clear dynamic pattern of microstates has been found. This study aims to identify the dynamics of EEG microstates in schizophrenia and to test whether schizophrenia patients with altered clinical symptoms severity showed different microstates abnormalities compared with healthy controls. Resting-state EEG data in 46 individuals who met the ICD-10 diagnostic criteria for schizophrenia and 39 healthy controls was recorded. The patients with schizophrenia were divided into subgroups based on the level of their negative or positive symptoms assessed using the Positive and Negative Syndrome Scale. Microstate parameters (contribution, occurrence, and duration) of four prototypical microstate classes (A–D) were investigated. Compared with healthy controls, individuals with schizophrenia showed increased duration and contribution of microstate class C, decreased contribution and occurrence of microstate class B. Different microstate patterns were found between subgroups and healthy controls. Results in this study support the consistent observation of abnormal EEG microstates patterns in patients with schizophrenia and highlight the necessity to divide subjects into subgroups according to their clinical symptoms.

## Introduction

Schizophrenia is a complex and devastating mental illness which has affected multiple aspects of patients. Despite numerous long-term studies, its pathogenesis still remains poorly understood. The synchronization models within or between numerous brain regions play an essential role in understanding the psychopathology of schizophrenia. Therefore, recent studies have been focusing on the involvement of the global brain functions in this disorder (1–3). To this end, the use of resting electroencephalogram (EEG) microstates is highly valued to investigate the endophenotypes for schizophrenia (4, 5). Microstates reflect the global brain function by instantly configuring the electrical field of the scalp (6); they remain stable for about 60–120 ms and quickly change into another class of microstates, and then become stable again, showing the semi-simultaneity of the brain network activity on a large scale (7). Besides, microstate sequences and the patterns of these sequences are related to the subsequent switching between these integrated states (7). Studies also reported that the microstates might be associated with different mental states and represent the collaborative activities in certain brain networks (8), therefore, they can be considered as the “atoms of thought.” A large body of evidence showed that the microstate time series could provide insight into the brain's neural activity at the rest state (9–11). Some studies using functional magnetic resonance imaging (fMRI) and EEG methods have shown significant correlations between microstate maps and fMRI resting-state networks (11). Although some studies argued that the attribution patterns of microstates to fMRI brain functions must be more complex (10), the microstates class A, B, C, and D were related closely with auditory, visual, saliency, and attention network, respectively (9). Therefore, microstate analysis of EEG is a helpful and powerful neurophysiological approach to study the global brain function; it is also inexpensive, and might be clinically translatable.

The microstates detected with EEG are highly replicable, and thus can be grouped into different sets according to their topographical similarity with the use of clustering algorithms. It is generally believed that the optimal number of microstate classes is closely related to the dataset studied. To date, there has been no consensus or unified standard on how to determine the optimal number of microstate classes (7). However according to pioneering studies, four major microstate classes, namely class A, B, C, and D, have been found. These four classes of microstates are highly consistent in resting-state EEG and can explain around 80% of the global variance of EEG data (12).

In some studies, patients with schizophrenia exhibited temporal dynamic abnormalities of EEG microstates, such as increased duration and occurrence of microstates class C (13, 14) and decreased duration and occurrence of microstates class D (15), as compared to healthy controls. A meta-analysis on studies of EEG microstates from 1999 to 2015 showed moderate alterations of two classes of microstates in patients with schizophrenia: higher frequency of microstate class C and shorter duration of microstate class D (16); there is also evidence for a slight shortening of microstate class B. Similar to the above study, a recent meta-analysis included studies published before November 29, 2019 (4) found that compared with healthy controls, individuals with schizophrenia showed consistently increased time coverage and occurrence of microstate class C, as well as decreased time coverage of microstate class D. Although the consistency regarding class C and D is remarkable, there are still inconsistent findings in studies (17, 18). As for microstate class A and B, the findings are inconsistent and more complicated (19–22).

A factor leading to the variation in previous findings might be the differences in clinical symptoms in patients with schizophrenia. Individuals with this disease often have different levels of positive or negative symptoms, which might be associated with different prognosis, cognition, medication, and psychophysiology (23, 24). Some studies have explored the relationship between specific abnormalities in EEG microstates and different clinical symptoms of schizophrenia. An early study in patients with chronic schizophrenia showed that the mean microstate duration was positively correlated with the total scores of the Scale for Assessment of Negative Symptoms and the Brief Psychiatric Rating Scale (25). According to some other studies, the duration of microstate class D was shorter in periods with hallucinations (26) and the degree of shortening was significantly correlated with the severity of paranoid hallucination (15). On the contrary, recent studies did not find any correlation between microstates and clinical symptoms in patients with chronic schizophrenia (4). Taken together, the above results might suggest a potential relationship between microstates and clinical symptoms. However, in previous studies, schizophrenia patients are usually considered as a unitary group and no conclusions could be drawn due to the inconsistent results. Therefore, studies on microstate patterns based on different symptoms of patients might provide insights for understanding the dynamics of microstates in schizophrenia.

To our knowledge, findings in previous researches did not propose a clear model for microstates in schizophrenia and inconsistent results may be related to different levels of psychopathological symptoms. In this study, to identify differences between schizophrenia patients and healthy controls with regard to microstates, we will first take the patients with schizophrenia as a unitary group. Further, we will divide the patients into subgroups based on the severity of their positive or negative symptoms, in order to investigate whether schizophrenia patients with altered clinical symptoms severity showed different microstates abnormalities compared with healthy controls.

## Materials and Methods

### Participants

Forty-six patients with schizophrenia (SCZ) aged between 18 and 60 years were enrolled in this study. All the patients were diagnosed with schizophrenia according to the ICD-10 criteria and the Mini-International Neuropsychiatric Interview. Thirty-nine healthy controls (HC) with matched gender and age were recruited from the community, according to the inclusion criterion of having no current or lifetime Axis I or II diagnoses. The exclusion criteria for all the participants were as follows: history of serious medical conditions, severe intellectual disability, previous episode of psychosis due to substance abuse, use of alcohol or benzodiazepine within 24 h, and inability to complete the test. Socio-demographic information such as age, gender, and education level was recorded for all the subjects. All the participants were fully informed of the procedures and signed the written informed consent form. The study was approved by the Clinical Research Ethics Committee of the Second Xiangya Hospital, Central South University.

To assess the clinical symptoms in schizophrenia patients, the Positive and Negative Syndrome Scale (PANSS) (27) was used. The PANSS scale consists of 30 items and is divided into three subscales, i.e., positive, negative, and general psychopathology subscales. All the items are scored with a 7-point scale. The 7 items in the subscale for positive symptoms were summed up to get a score for positive symptoms, which ranged from 7 to 49; the score for negative symptoms was calculated in the same way.

Patients with schizophrenia were classified as having high or low level of positive symptoms according to the median of the overall severity of positive symptoms (Median = 15.5, Range = 7–30); patient with a higher score for positive symptoms than the median were assigned into the group with high level of positive symptoms (HP) and those with a score lower than or equal to the median were assigned into the group with low level of positive symptoms (LP). Similarly, patients were assigned into the group with high level of negative symptoms (HN) or the group with low level of negative symptoms (LN), according to the median of the overall severity of negative symptoms (Median = 12, Range = 7–33). After classification, there were 23 patients in the HP group, 23 patients in the LP group 26 in the LN group and 20 in the HN group.

Among the 46 patients, 20 were receiving antipsychotic medication; there was no difference in the medication status between the subgroups (χ2 = 0.354, *p* = 0.552, χ2 = 0.174, *p* = 0.676; when divided according to positive and negative symptoms respectively). No significant correlation between positive and negative symptoms scores were found (*r* = −0.087, *p* = 0.565).

### EEG Recordings

The EEG data were acquired using a 64 BrainAmp cap (BrainProducts GmbH, Munich, Germany), with electrodes positioned according to the 10–20 International System. An additional electrode was used as the ground. The linked mastoid (TP9 and TP10) served as the reference for all the electrodes. The vertical electro-oculogram (VEOG) was recorded from the electrode below the right eye. The signals recorded were filtered with a bandpass of 0.1–1,000 Hz, and all impedances of the electrodes were kept below 10 kΩ. During the recording, the participants was instructed to sit comfortably in a chair, keep relaxed with eyes closed.

### Data Pre-processing

Offline pre-processing was performed using the software EEGLAB. The VEOG channel were removed, therefore 61 channels were retained left for the further analyses. The EEG data were filtered with a bandpass of 0.1–70 Hz and then with a notch filter of 48–52 Hz. Bad EEG periods were removed through visual inspection, and interpolation of bad channels with severe artifacts across the whole recording. The data were then divided into 2 s segments and an infomax-based independent component analysis (ICA) was conducted with residual eye- and muscular- artifacts were removed (28). Finally, the data were re-referenced to the common average reference and filtered with a bandpass of 2–20 Hz.

### Microstate Analysis

Microstate analysis was performed with the Microstate Analysis plugin developed by Thomas Koenig (http://www.thomaskoenig.ch/index.php/software/). Individual microstate maps for each subject were calculated from original momentary maps. To extract EEG microstates, the peaks of the global field power (GFP) were firstly extracted, and topographic maps occurring at the peaks of the GFP curve were then submitted to a modified k-means clustering algorithm to isolate map topographies. According to the most common (12) and reproducible (7) classification, the number of microstates classes were defined as four. The number of repetitions was set at 20 and the maximum number of iterations was set at infinite. The group-level microstate classes were then identified for SCH and HC patients separately. Using the mean microstate classes across all the participants as the template, individual and group-level maps were sorted out, and the following parameters were extracted for the four microstate classes: globally explained variance, contribution (the proportion of time spent for each microstate), occurrence (the total number of the microstate of a given class per second), and duration (the mean duration of a microstate class in milliseconds).

### Statistics Analyses

For continuous variables, inter-group comparisons were performed using *t-test*. Gender difference between group was tested using Pearson's χ2 test. Inter-group differences in microstate parameters between SCZ subgroups or between SCZ patients and HC were tested using repeated measure analysis of variance (rm-ANOVA), with group (SCZ or LP or HP or LN or HN and HC) as between-subject factor, and microstate classes (A-D) and microstate parameters (contribution, occurrence, and duration) as within-subject factors. The Greenhouse-Geisser correction was applied for multiple comparisons. *Post hoc* tests were performed only when statistical significance was indicated in the rm-ANOVA. Pairwise inter-group comparisons for microstate classes and parameters were corrected for multiple comparisons with Bonferroni correction. All the analyses were conducted using the SPSS Version 23.0.

## Results

### Subject Characteristics

The demographic and clinical characteristics of SCZ patients and HC are presented in Table 1. There was no significant difference in gender, age and education level between groups. There was no significant difference in the score of PANSS negative symptoms between the LP and HP subgroups (Supplementary Table 1), and no significant difference in the score of PANSS positive symptoms between the LN and HN subgroups (Supplementary Table 2). PANSS general psychopathology significantly differed between the LP and HP subgroups as well as between the LN and HN subgroups, with HP group showed higher PANSS general psychopathology score than LP group, and HN group showed higher PANSS general psychopathology score than LN group.

**Table 1:** Demographic and clinical characteristics of all the participants.

	**HC (***n*** = 39)**	**SCZ (***n*** = 46)**	***t*** **/ χ2**	***P***
Gender (M/F)	25/14	36/10	2.088	0.148
Age (years, mean ± SD)	27.21 ± 6.92	28.72 ± 7.44	0.964	0.338
Education (years, mean ± SD)	14.08 ± 2.28	13.16 ± 3.01	1.588	0.116
Age at onset (years, mean ± SD)		24.64 ± 6.93		
Medication (yes/no)		20/23		
Illness duration (years, mean ± SD)		4.11 ± 5.82		
PANSS positive		15.91 ± 5.86		
PANSS negative		14.41 ± 7.74		
PANSS general psychopathology		30.98 ± 7.38		
PANSS total		61.30 ± 16.28		

*SCH, individuals with schizophrenia; HC, healthy controls; PANSS, Positive and Negative Syndrome Scale*.

### Data Quality

After the rejection of artifacts, the numbers of 2-sec segments included in the analysis for each group was 100.10 ± 36.358 for HC, 94.8 ± 24.414 for patients with SCH, 97.87 ± 21.663 for the HP group, 91.74 ± 27.022 for the LP group, 99.60 ± 25.525 for the HN group, and 91.12 ± 23.348 for the HN group.

### Microstate Parameters: Overall Results

**Figure 1:**
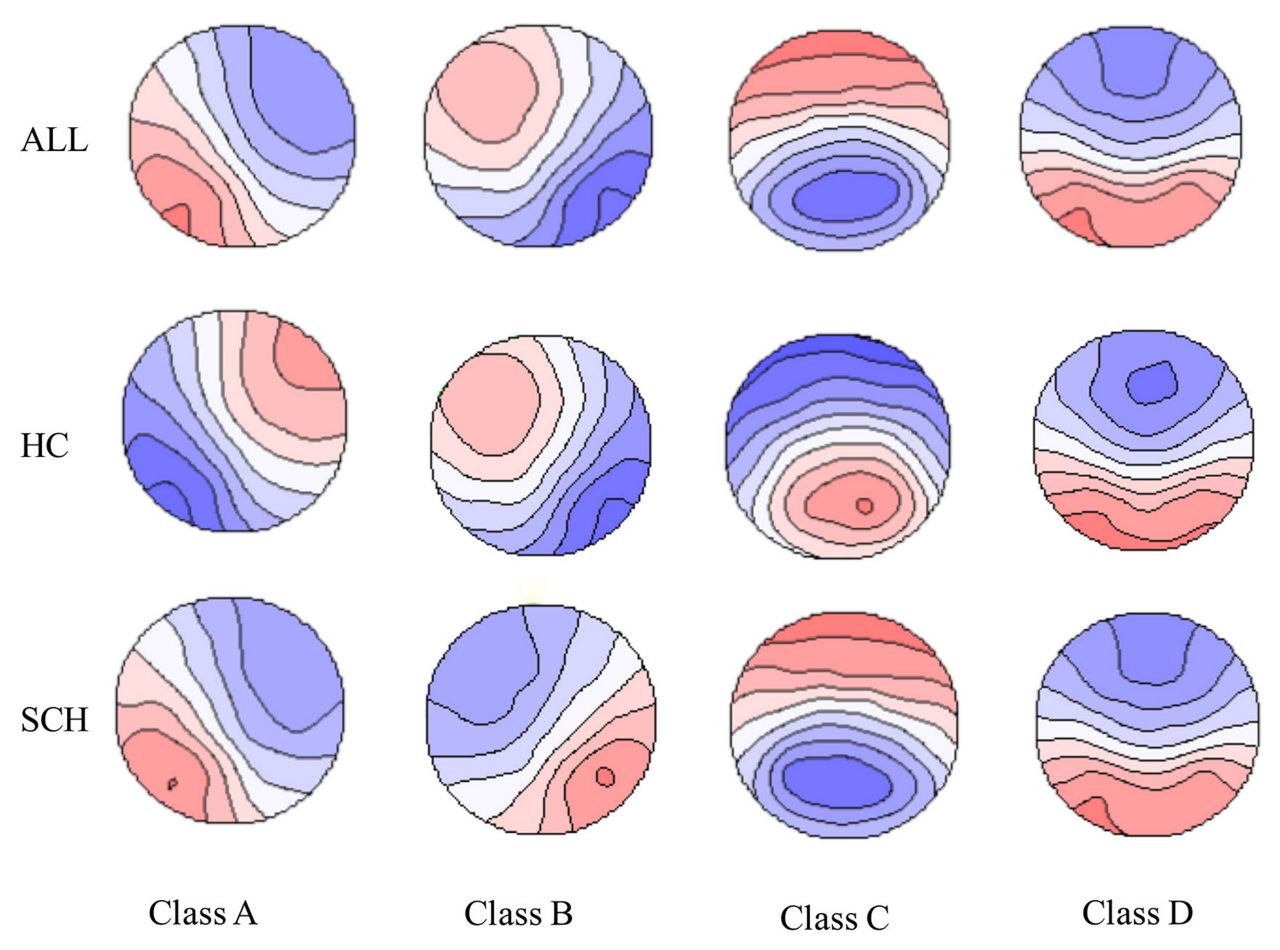
The spatial configuration of the four microstate claasses for the two groups and all the participants. Polarity was ignored. SCH, individuals with schizophrenia; HC, healthy controls; ALL, all the participants in this study.

The overall maps, and the maps for patients with SCH and HC are shown in Figure 1. The spatial configuration of the four microstate classes for each subgroup was presented in Supplementary Figures 1, 2. The four microstate classes explained 76 and 78% of the global variance for the SCH group and the HC, respectively. For each subgroup, the four microstate classes explained 78% of the global variance in the HP group, and the percentage was 77% in the LP group, 77% in the HN group, and 79% in the LN group.

### Inter-group Differences in Microstate Parameters

**Figure 2:**
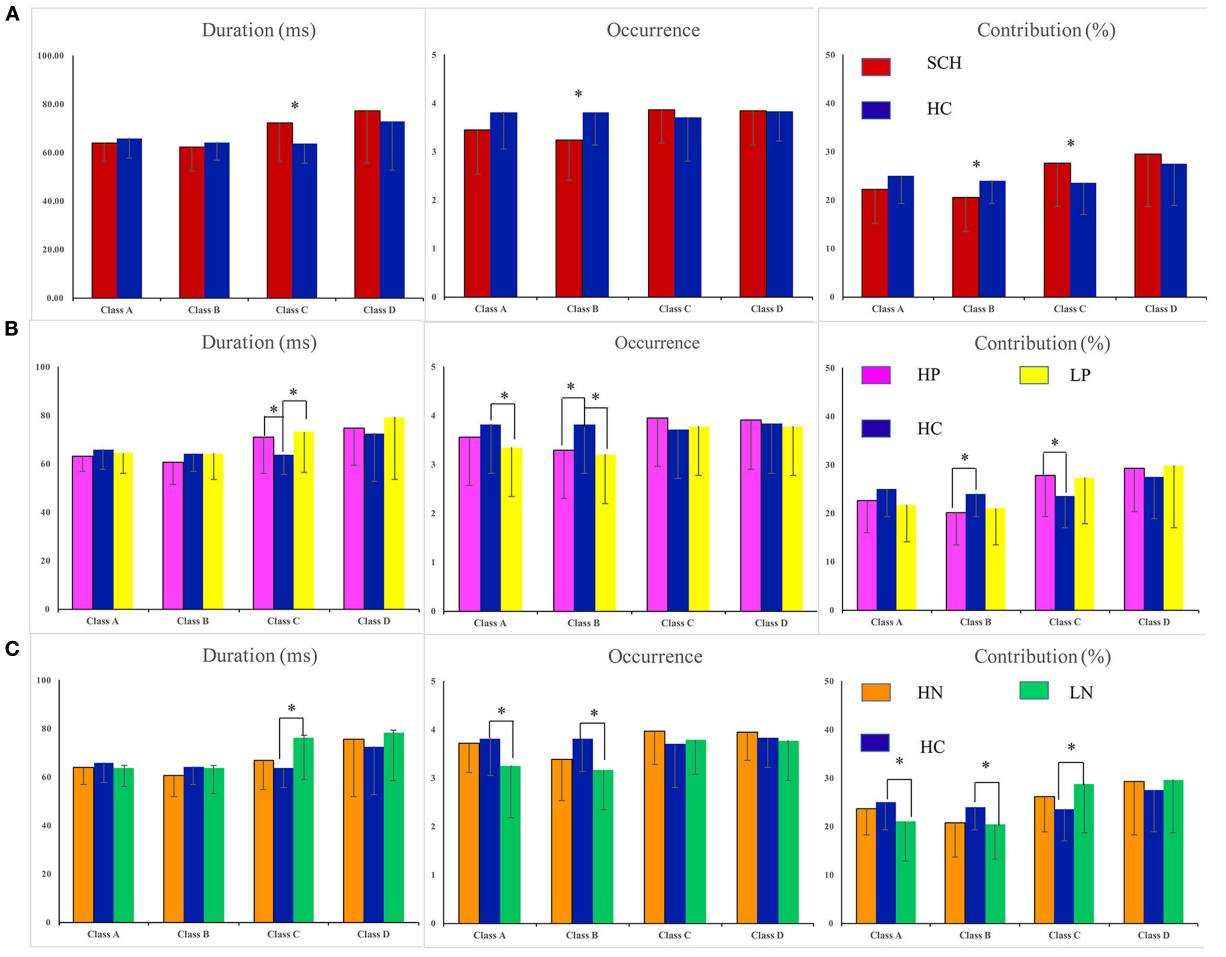
Statistics of the microstate parameters: duration, contribution, and occurrence. *P < 005. Errror bars represent standard error. SCH, individuals with schizophrenia; HC, healthy controls; HP, schizophrenia patients with high levels of postive symptoms; LP, schizophrenia patients with low levels of postive symptoms; HN, schizophrenia patients with high level of negative symptoms; LN, schizophrenia patients with low lwvwl of negative symptoms. **(A)** SCH vs. HC. **(B)** Comparisions were made between groups (HP vs. HC, LP vs. HC). **(C)** Comparisons were made between groups (HN vs. HC, LN vs. HC).

The four parameters for SCH, subgroups (HP, LP, HN, LN) and healthy controls are presented in Figure 2. For SCH vs. HC, main effect was found (F = 4.177, *p* = 0.044), and rm-ANOVA showed an interaction of group × microstate parameters × microstate class [*F*_(6, 498)_ = 4.008, *p* = 0.009]. *Post hoc* analysis revealed that the interaction effect was related to differences in microstate class B and class C. Compared with HC, patients with SCH showed increased mean duration and contribution of microstate class C and decreased mean contribution and occurrence of microstate class B. No statistically significant inter-group difference was found for microstate class A and D. The means and standard deviations for all considered parameters and microstate classes are reported in Supplementary Table 3. Detailed results of rm-ANOVA are presented in Supplementary Table 4 and *Post hoc* results in Supplementary Table 5.

For HP vs. HC, an interaction of group × microstate parameters × microstate class [*F*_(6, 360)_ = 2.860, *p* = 0.049] was found. *Post hoc* analysis revealed that, compared with HC, the HP group showed increased duration and contribution of microstate class C and decreased occurrence and contribution of microstate class B (Figure 2B). Detailed results of rm-ANOVA and *Post hoc* results for HP vs. HC are presented in Supplementary Tables 6, 7.

For LP vs. HC, an interaction of group × microstate parameters [*F*_(6, 120)_ = 4.059, *p* = 0.048] and an interaction of microstate parameters × microstate class [*F*_(6, 360)_ = 8.224, *p* = 0.001] were found. *Post hoc* analysis showed that the LP group had decreased occurrence of microstate class A and B, and increased duration of microstate class C, as compared with HC (Figure 2B). Detailed results of rm-ANOVA and *Post hoc* results for LP vs. HC are presented in Supplementary Tables 8, 9.

For LN vs. HC, rm-ANOVA revealed an interaction of group × microstate parameters × microstate class [*F*_(6, 378)_ = 4.155, *p* = 0.015]. Simple effect analysis revealed that compared with HC, LN group showed decreased occurrence and contribution of microstate class A and B, and increased duration and contribution of microstate class C (Figure 2C). Detailed results of rm-ANOVA and *Post hoc* results for LN vs. HC are presented in Supplementary Tables 11, 12.

For HN vs. HC, no main effect or interaction was found (Figure 2C). Detailed results of rm-ANOVA for HN vs. HC are presented in Supplementary Table 10.

## Discussion

In this study, we have found that patients with SCH showed increased duration and contribution of microstate class C, decreased contribution and occurrence of microstate class B. Nevertheless, different patterns were found when we divided the SCH patients into subgroups according to the level of positive and negative symptoms. Specifically, both HP and LP showed increased duration and contribution of microstate class C, decreased occurrence and contribution of microstate class B, as compared to HC. Besides, the LP group also showed decreased occurrence and contribution of microstate class A. The LN group showed increased duration of microstate class C, decreased occurrence of microstate class A and B, but no difference was found between the HN group and HC group.

In patients with schizophrenia, altered temporal dynamics of EEG microstates had been found in several studies (4, 15, 21). Similar patterns were also found in high-risk populations of psychosis (18, 22), such as siblings of schizophrenia patients (4). Consistent with these studies, our findings demonstrated altered microstate class C in SCH as well as most subgroups. As patients with schizophrenia usually exhibit abnormal assignment of saliency (29) as well as dysfunction of attentional processing and executive control (30, 31), the increased occurrence of microstate class C in schizophrenia might be a sign of imbalance across processes involved in saliency. A fMRI-EEG study suggested that microstate class C was correlated with the cerebral activations in the posterior part of the anterior cingulate gyrus, the left claustrum, bilateral inferior frontal gyrus, as well as the right anterior insula (9), which have been found to be part of the saliency-network (32, 33) and to play a critical role in switching between the default mode and executive function mode (34).

Previous studies found a decrease in microstate class B in medication-free schizophrenia patients, as compared to healthy controls (20, 35). In addition, a study found that microstate class B could be used to distinguish patients with first-episode psychosis from high-risk individuals with and without later transition to psychosis (36) and proposed that microstate class B might be a state biomarker underlying the progression of psychosis. However, some studies had shown the opposite effect (18, 37) in medicated patients, which was attributed to the effect of antipsychotic drugs on microstates. This effect can also be used to explain our findings, since more than half of the subjects with schizophrenia were medication-free. As for microstate class A, inconsistent and complicated results have been yielded. Similar to a recent study on first-episode psychosis (21), our study also identified decreased occurrence of microstate class A, while some other studies found an increase (18, 20). No consistent inter-group difference between patients with schizophrenia and healthy controls was found for microstate class A in a recent meta-analysis (4).

We did not find any abnormalities in microstate class D in patients with SCZ, which was inconsistent with literature reports (4). But this difference could explain by medication, as antipsychotic drugs might have a normalization effect on microstate dynamics (i.e., increasing the occurrence of microstate class D) (14). Besides, from a general perspective, the maps assigned to a certain microstate class varied in different studies, especially for microstate classes C and D (12), which might be one of the reasons for the inconsistencies between the present study and some provious studies in terms of microstate class D in patients with SCZ.

In this study, we observed different microstate patterns when compared subgroups and healthy controls, although it is difficult to explain the functional significance of microstate changes showed in these subgroups, it highlighted the necessity to distinguish patients according to their clinical symptoms. Our findings also provided another explanation for the inconsistent results in studies on microstates in patients with schizophrenia. In future works, patients with schizophrenia could be grouped on the basis of their clinical symptoms, in order to reduce the heterogeneity of subjects and obtain results which may be more consistent.

One of the main limitations of this study is the psychometric measure we used to divide schizophrenia patients into different subgroups. The results are likely to be different if we used another scale such as the Brief Psychiatric Rating Scale. In addition, individuals with high levels of both positive and negative symptoms may show different microstate patterns compared to those with high levels of positive or negative symptoms alone. However, we were only able to investigate positive or negative symptoms independently by dividing the SCZ patients according to the subscale scores of PANSS. Treatments, especially drug therapy, are highly likely to affect the microstate parameters. In this study, some patients were on medication, which might be one of the reasons for some differences observed in this study. Another limitation in this study is that four microstate classes were selected as they had been established in most previous studies (6). Although the four microstate classes explained more than 76% of the variance for each group, it is possible that the abnormalities in SCZ patients are undetected in the remaining 20% of the components. Nevertheless, the four microstate classes allowed direct comparison between this study and previous researches. Lastly, with the relatively small number of patients, especially the small number of subjects included in each subgroup, the results might be less accurate due to sampling error.

In summary, our results suggested that patients with schizophrenia have abnormal EEG microstates, especially the microstate class C. However, different patterns were found when we divided the schizophrenia patients into subgroups according to the level of positive and negative symptoms, which may suggest different neural mechanisms underling positive and negative symptoms and highlight the necessity to differentiate patients according to their clinical symptoms.

## Data Availability Statement

The original contributions presented in the study are included in the article/Supplementary Material, further inquiries can be directed to the corresponding author/s.

## Ethics Statement

The studies involving human participants were reviewed and approved by Clinical Research Ethics Committee of The Second Xiangya Hospital, Central South University. The patients/participants provided their written informed consent to participate in this study.

## Author Contributions

XW and JZ conceived and design the research, and revised the paper. QS contributed to the data collection and analysis and wrote the first draft of the manuscript. HG, NG, RL, YH, and WG contributed to the data collection. All authors have approved the final manuscript.

## Funding

This work was supported by the National Natural Science Foundation of China (82071543), Science and Technology Program of Hunan Provience (2019SK2334), and Natural Science Foundation of Hunan Province (2019JJ40424).

## Conflict of Interest

The authors declare that the research was conducted in the absence of any commercial or financial relationships that could be construed as a potential conflict of interest.

## Publisher's Note

All claims expressed in this article are solely those of the authors and do not necessarily represent those of their affiliated organizations, or those of the publisher, the editors and the reviewers. Any product that may be evaluated in this article, or claim that may be made by its manufacturer, is not guaranteed or endorsed by the publisher.
