# Supplementary material for: EEG Microstates and Its Relationship With Clinical Symptoms in Patients With Schizophrenia
Source: Front Psychiatry. 2021 Oct 28;12:761203. doi: 10.3389/fpsyt.2021.761203 (PMC8581189; doi:10.3389/fpsyt.2021.761203)
Supplement: Supplementary file 1 [file Data_Sheet_1.docx]

Supplementary Figures

**
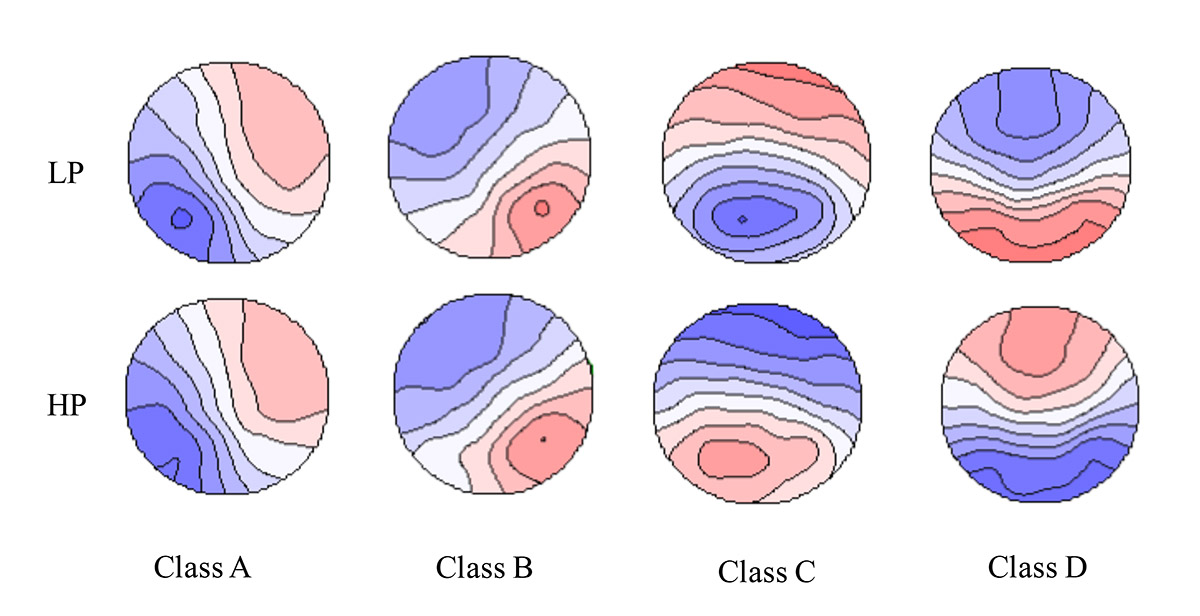
**

**Supplementary Figure 1.** The spatial configuration of the four microstate classes for two subgroups according to positive symptoms. HP, schizophrenia patients with high levels of positive symptoms; LP, schizophrenia patients with low levels of positive symptoms.

**
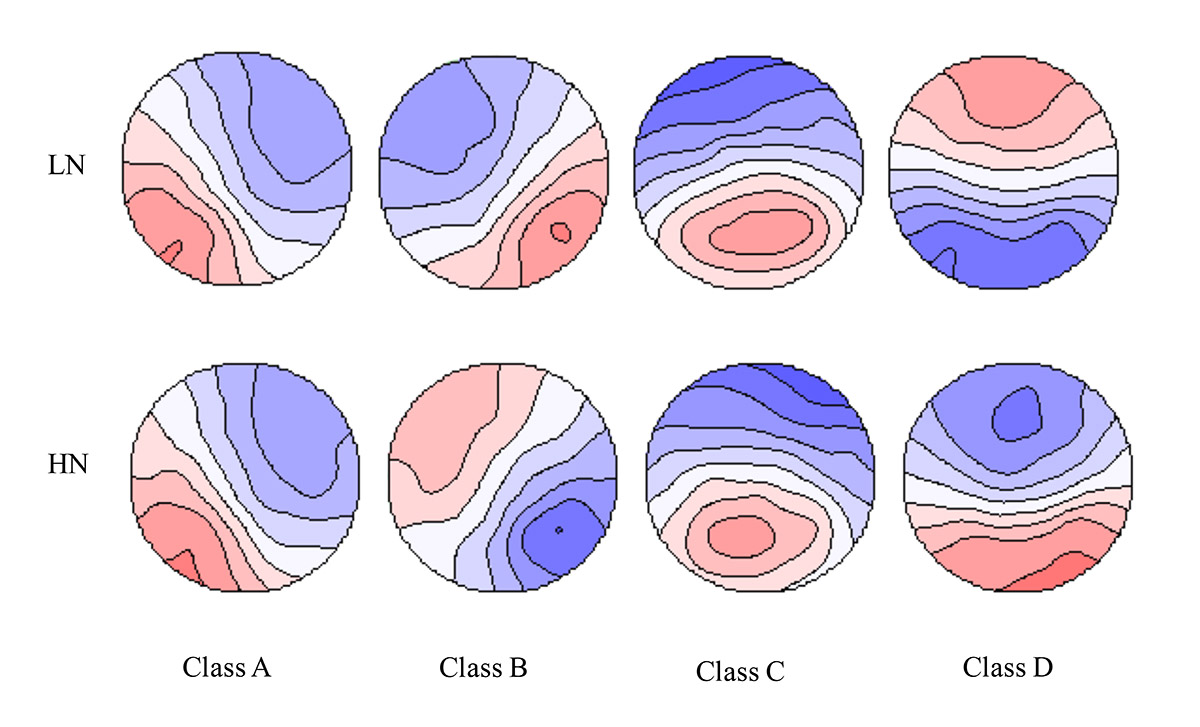
**

**Supplementary Figure 2**. The spatial configuration of the four microstate classes for two subgroups according to negative symptoms. HN, schizophrenia patients with high levels of negative symptoms; LN, schizophrenia patients with low levels of negative symptoms.
