# Supplementary material for: EEG Microstates and Its Relationship With Clinical Symptoms in Patients With Schizophrenia
Source: Front Psychiatry. 2021 Oct 28;12:761203. doi: 10.3389/fpsyt.2021.761203 (PMC8581189; doi:10.3389/fpsyt.2021.761203)
Supplement: Supplementary file 2 [file Table_1.docx]

Supplementary Tables

**Supplementary Table 1**. Demographic and clinical characteristics of two subgroups according to positive symptoms.

|  | HP (n = 23) | LP (n = 23) | t/ χ2 | P |
| --- | --- | --- | --- | --- |
| Gender (M/F) | 17/6 | 19/4 | 0.511 | 0.475 |
| Age (years, mean ± SD) | 29.96 ± 7.46 | 27.48 ± 7.38 | 1.133 | 0.264 |
| Education (years, mean ± SD) | 13.35 ± 3.38 | 12.95 ± 2.63 | 0.434 | 0.666 |
| Age at onset (years, mean ± SD) | 25.17 ± 7.08 | 24.09 ± 6.90 | 0.520 | 0.606 |
| Medication (yes/no) | 9/14 | 11/12 | 0.884 | 0.381 |
| Illness duration (years, mean ± SD) | 4.87 ± 6.92 | 3.34 ± 4.59 | 0.354 | 0.552 |
| PANSS positive | 20.61 ± 3.91 | 11.22 ± 2.97 | 9.181 | < 0.001 |
| PANSS negative | 15.26 ± 7.51 | 13.56 ± 8.04 | 0.739 | 0.464 |
| PANSS general psychopathology | 33.78 ± 8.14 | 28.17 ± 5.36 | 2.760 | 0.008 |

HP, schizophrenia patients with high levels of positive symptoms; LP, schizophrenia patients with low levels of positive symptoms; PANSS, Positive and Negative Syndrome Scale.

**Supplementary Table 2.** Demographic and clinical characteristics of two subgroups according to negative symptoms.

|  | HN (n = 20) | LN (n = 26) | t/ χ2 | P |
| --- | --- | --- | --- | --- |
| Gender (M/F) | 16/4 | 20/6 | 0.063 | 0.547 |
| Age (years, mean ± SD) | 29.45 ± 8.19 | 28.15 ± 6.93 | 0.581 | 0.564 |
| Education (years, mean ± SD) | 12.35 ± 2.76 | 13.80 ± 3.10 | -1.638 | 0.109 |
| Age at onset (years, mean ± SD) | 24.85 ± 7.18 | 24.48 ± 6.87 | 0.176 | 0.861 |
| Medication (yes/no) | 8/12 | 12/14 | 0.557 | 0.581 |
| Illness duration (years, mean ± SD) | 4.65 ± 5.97 | 3.68 ± 5.79 | 0.174 | 0.676 |
| PANSS positive | 16.35 ± 5.67 | 15.58 ± 6.09 | 0.440 | 0.662 |
| PANSS negative | 21.55 ± 6.36 | 8.92 ± 2.24 | 8.48 | < 0.001 |
| PANSS general psychopathology | 34.05 ± 7.96 | 28.62 ± 6.04 | 2.634 | 0.012 |

HN, schizophrenia patients with high levels of negative symptoms; LN, schizophrenia patients with low levels of negative symptoms; PANSS, Positive and Negative Syndrome Scale.

**Supplementary Table 3.** Group means and standard deviations for all considered parameters and microstate classes

| MS | | Group | | | | | |
| --- | --- | --- | --- | --- | --- | --- | --- |
|  |  | HC | SCH | HP | LP | HN | LN |
| Duration (ms) | A | 65.94 ± 8.25 | 63.85 ± 7.34 | 63.15 ± 6.16 | 64.56 ± 8.44 | 63.99 ± 7.04 | 63.75 ± 7.70 |
|  | B | 64.23 ± 7.46 | 62.43 ± 9.94 | 60.48 ± 8.78 | 64.39 ± 10.82 | 60.57 ± 8.63 | 63.87 ± 10.78 |
|  | C | 63.62 ± 7.98 | 72.13 ± 15.69 | 71.03 ± 14.76 | 73.23 ± 16.84 | 66.81 ± 12.09 | 76.22 ± 17.09 |
|  | D | 72.73 ± 19.80 | 77.16 ± 21.30 | 74.95 ± 15.68 | 79.36 ± 25.92 | 75.49 ± 23.56 | 78.44 ± 19.75 |
| Occurrence | A | 3.82 ± 0.76 | 3.46 ± 0.93 | 3.57 ± 0.90 | 3.35 ± 0.95 | 3.72 ± 0.60 | 3.26 ± 1.08 |
|  | B | 3.81 ± 0.67 | 3.25 ± 0.83 | 3.30 ± 0.89 | 3.21 ± 0.79 | 3.38 ± 0.84 | 3.16 ± 0.82 |
|  | C | 3.71 ± 0.90 | 3.87 ± 0.70 | 3.96 ± 0.67 | 3.78 ± 0.73 | 3.98 ± 0.69 | 3.79 ± 0.71 |
|  | D | 3.83 ± 0.61 | 3.85 ± 0.72 | 3.91 ± 0.61 | 3.78 ± 0.84 | 3.94 ± 0.57 | 3.78 ± 0.83 |
| Contribution (%) | A | 24.99 ± 5.70 | 22.21 ± 7.12 | 22.71 ± 6.71 | 21.71 ± 7.63 | 23.76 ± 5.44 | 21.02 ± 8.09 |
|  | B | 24.03 ± 4.67 | 20.60 ± 7.03 | 20.17 ± 6.72 | 21.03 ± 7.46 | 20.78 ± 7.01 | 20.46 ± 7.19 |
|  | C | 23.49 ± 6.56 | 27.64 ± 9.00 | 27.88 ± 8.59 | 27.40 ± 9.58 | 26.13 ± 7.15 | 28.81 ±1 0.18 |
|  | D | 27.50 ± 8.64 | 29.54 ± 10.86 | 29.23 ± 8.82 | 29.85 ± 12.78 | 29.33 ± 11.03 | 29.71 ± 10.94 |

SCZ, individuals with schizophrenia; HC, healthy controls; HP, schizophrenia patients with high levels of positive symptoms; LP, schizophrenia patients with low levels of positive symptoms; HN, schizophrenia patients with high levels of negative symptoms; LN, schizophrenia patients with low levels of negative symptoms.

**Supplementary Table 4**. Detailed results of the rm-ANOVA on microstate temporal parameters for SCH and HC group.

| rm-ANOVA | F | P |
| --- | --- | --- |
| PAR | 6238.379 | < 0.001 |
| PAR × Group | 4.125 | 0.045 |
| MS | 3.404 | 0.020 |
| MS × Group | 4.013 | 0.009 |
| PAR × MS | 2.690 | 0.049 |
| PAR × MS × Group | 4.008 | 0.009 |

MS, microstate class; PAR, microstate parameter; SCH, individuals with schizophrenia; HC, healthy controls. The Greenhouse-Geisser correction for multiple comparisons was applied.

**Supplementary Table 5.** Post-hoc group comparisons (HC vs. SCH of all microstate parameters (mean duration, contribution, and occurrence), Benferroni corrected, for each microstate class (A, B, C, and D).

| Microstate parameters | Microstate classes | SCH vs. HC | | |
| --- | --- | --- | --- | --- |
|  |  | p | d | 95%CI |
| Duration (ms) | A | 0.221 | -2.084 | -5.447, 1.279 |
|  | B | 0.356 | -1.796 | -5.645, 2.054 |
|  | **C** | **0.003** | **8.515** | **2.991, 14.038** |
|  | D | 0.327 | 4.426 | -4.505, 13.356 |
| Occurrence/s | A | 0.059 | -0.356 | -0.726, 0.015 |
|  | B | **0.001** | **-0.554** | **-0.883, -0.224** |
|  | C | 0.347 | 0.164 | -0.181, 0.509 |
|  | D | 0.888 | 0.021 | -0.271, 0.312 |
| Contribution (%) | A | 0.053 | -2.778 | -5.597, 0.041 |
|  | B | **0.011** | **-3.430** | **-6.057, -0.802** |
|  | C | **0.019** | **4.151** | **0.698, 7.604** |
|  | D | 0.345 | 2.047 | -2.242, 6.336 |

SCZ, individuals with schizophrenia; HC, healthy controls.

**Supplementary Table 6.** Detailed results of the rm-ANOVA on microstate temporal parameters for HP and HC group

| rm-ANOVA | F | P |
| --- | --- | --- |
| PAR | 3366.169 | < 0.001 |
| PAR × Group | 0.187 | 0.667 |
| MS | 9.010 | < 0.001 |
| MS × Group | 3.159 | 0.040 |
| PAR × MS | 10.924 | < 0.001 |
| PAR × MS × Group | 2.860 | 0.049 |

MS, microstate class; PAR, microstate parameter; HP, schizophrenia patients with high levels of positive symptoms; HC, healthy controls. The Greenhouse-Geisser correction for multiple comparisons was applied.

**Supplementary Table 7.** Post-hoc group comparisons (HP vs. HC) of all microstate parameters (mean duration, contribution, and occurrence), Benferroni corrected, for each microstate class (A, B, C, and D).

| Microstate parameters | Microstate classes | HP vs. HC | | |
| --- | --- | --- | --- | --- |
|  |  | p | d | 95%CI |
| Duration (ms) | A | 0.165 | -2.791 | -6.760, 1.179 |
|  | B | 0.079 | -3.750 | -7.942, 0.443 |
|  | C | **0.013** | **7.417** | **1.649, 13.185** |
|  | D | 0.648 | 2.217 | -7.459, 11.893 |
| Occurrence/s | A | 0.266 | -0.242 | -0.672, 0.188 |
|  | B | **0.013** | **-0.509** | **-0.907, -0.111** |
|  | C | 0.245 | 0.254 | -0.179, 0.686 |
|  | D | 0.601 | 0.084 | -0.235, 0.403 |
| Contribution (%) | A | 0.160 | -2.277 | -5.480, 0.927 |
|  | B | **0.010** | **-3.857** | **-6.755, -0.958** |
|  | C | **0.027** | **4.390** | **0.515, 8.266** |
|  | D | 0.451 | 1.738 | -2.842, 6.317 |

HP, schizophrenia patients with high levels of positive symptoms; HC, healthy controls.

**Supplementary Table 8.** Detailed results of the rm-ANOVA on microstate temporal parameters for LP and HC group

| rm-ANOVA | F | P |
| --- | --- | --- |
| PAR | 3507.794 | < 0.001 |
| PAR × Group | 4.059 | 0.048 |
| MS | 7.155 | 0.001 |
| MS × Group | 2.266 | 0.112 |
| PAR × MS | 8.224 | 0.001 |
| PAR × MS × Group | 1.917 | 0.155 |

MS, microstate class; PAR, microstate parameter; LP, schizophrenia patients with low levels of positive symptoms; HC, healthy controls. The Greenhouse-Geisser correction for multiple comparisons was applied.

**Supplementary Table 9.** Post-hoc group comparisons (LP vs. HC) of all microstate parameters (mean duration, contribution, and occurrence), Benferroni corrected, for each microstate class (A, B, C, and D).

| Microstate parameters | Microstate classes | LP vs. HC | | |
| --- | --- | --- | --- | --- |
|  |  | p | d | 95%CI |
| Duration  (ms) | A | 0.531 | -1.378 | -5.752, 2.996 |
|  | B | 0.946 | 0.159 | -4.491, 4.809 |
|  | C | **0.003** | **9.612** | **3.294, 15.931** |
|  | D | 0.261 | 6.634 | -5.062, 18.331 |
| Occurrence/s | A | **0.037** | **-0.470** | **-0.911, -0.029** |
|  | B | **0.002** | **-0.598** | **-0.974, -0.222** |
|  | C | 0.736 | 0.075 | -0.367, 0.517 |
|  | D | 0.818 | -0.042 | -0.410, 0.325 |
| Contribution(%) | A | 0.059 | -3.279 | -6.686, 0.127 |
|  | B | 0.056 | -3.003 | -6.081, 0.076 |
|  | C | 0.061 | 3.912 | -0.191, 8.015 |
|  | D | 0.390 | 2.356 | -3.089, 7.801 |

LP, schizophrenia patients with low levels of positive symptoms; HC, healthy controls.

**Supplementary Table 10.** Detailed results of the rm-ANOVA on microstate temporal parameters for HN and HC group.

| rm-ANOVA | F | P |
| --- | --- | --- |
| PAR | 3238.704 | < 0.001 |
| PAR × Group | 0.003 | 0.956 |
| MS | 7.152 | 0.002 |
| MS × Group | 1.245 | 0.269 |
| PAR × MS | 8.622 | 0.001 |
| PAR × MS × Group | 0.921 | 0.392 |

MS, microstate class; PAR, microstate parameter; HN, schizophrenia patients with high levels of negative symptoms; HC, healthy controls. The Greenhouse-Geisser correction for multiple comparisons was applied. Group main effect (F = 0.001, *p* = 0.980) was not significant. Therefore, no post hoc analysis was applied.

**Supplementary Table 11.** Detailed results of the rm-ANOVA on microstate temporal parameters for LN and HC group.

| rm-ANOVA | F | P |
| --- | --- | --- |
| PAR | 3670.938 | < 0.001 |
| PAR × Group | 4.686 | 0.034 |
| MS | 9.155 | < 0.001 |
| MS × Group | 4.304 | 0.013 |
| PAR × MS | 10.648 | < 0.001 |
| PAR × MS × Group | 4.155 | 0.015 |

MS, microstate class; PAR, microstate parameter; LN, schizophrenia patients with low levels of negative symptoms; HC, healthy controls. The Greenhouse-Geisser correction for multiple comparisons was applied.

**Supplementary Table 12.** Post-hoc group comparisons (HC vs. LN) of all microstate parameters (mean duration, contribution, and occurrence), Benferroni corrected, for each microstate class (A, B, C, and D).

| Microstate parameters | Microstate classes | LN vs. HC | | |
| --- | --- | --- | --- | --- |
|  |  | p | d | 95%CI |
| Duration  (ms) | A | 0.286 | -2.188 | -6.253, 1.876 |
|  | B | 0.873 | -0.363 | -4.881, 4.155 |
|  | **C** | **< 0.001** | **12.609** | **6.322, 18.896** |
|  | D | 0.259 | 5.712 | -4.300, 15.723 |
| Occurrence/s | A | **0.017** | **-0.559** | **-1.017, -0.101** |
|  | B | **0.001** | **-0.651** | **-1.023, -0.279** |
|  | C | 0.701 | 0.081 | -0.339, 0.501 |
|  | D | 0.790 | -0.048 | -0.404, 0.308 |
| Contribution(%) | A | **0.024** | **-3.965** | **-7.382, -0.548** |
|  | B | **0.018** | **-3.567** | **-6.503, -0.631** |
|  | C | **0.013** | **5.319** | **1.175, 9.464** |
|  | D | 0.368 | 2.211 | -2.657, 7.080 |

LN, schizophrenia patients with low levels of negative symptoms; HC, healthy controls.
